# HLA-G Dimers in the Prolongation of Kidney Allograft Survival

**DOI:** 10.1155/2014/153981

**Published:** 2014-03-30

**Authors:** Maureen Ezeakile, Vera Portik-Dobos, Juan Wu, Daniel D. Horuzsko, Rajan Kapoor, Muralidharan Jagadeesan, Laura L. Mulloy, Anatolij Horuzsko

**Affiliations:** ^1^Molecular Oncology and Biomarkers Program, Cancer Center, Department of Medicine, Georgia Regents University, 1410 Laney Walker Boulevard, Augusta, GA 30912, USA; ^2^Section of Nephrology, Hypertension, and Transplantation Medicine, Department of Medicine, Georgia Regents University, Augusta, GA 30912, USA

## Abstract

Human leukocyte antigen-G (HLA-G) contributes to acceptance of allografts in solid organ/tissue transplantation. Most studies have determined that soluble HLA-G isoforms are systematically detected in serum/plasma of transplanted patients with significantly fewer episodes of acute and/or chronic rejection of allogeneic tissue/organ. Current models of the interactions of HLA-G and its specific receptors explain it as functioning in a monomeric form. However, in recent years, new data has revealed the ability of HLA-G to form disulfide-linked dimeric complexes with high preferential binding and functional activities. Limited data are available on the role of soluble HLA-G dimers in clinical pathological conditions. We describe here the presence of soluble HLA-G dimers in kidney transplant patients. Our study showed that a high level of HLA-G dimers in plasma and increased expression of the membrane-bound form of HLA-G on monocytes are associated with prolongation of kidney allograft survival. We also determined that the presence of soluble HLA-G dimers links to the lower levels of proinflammatory cytokines, suggesting a potential role of HLA-G dimers in controlling the accompanying inflammatory state.

## 1. Introduction

Human leukocyte antigen-G (HLA-G) is a natural molecule involved in the establishment and maintenance of maternal tolerance to semiallogeneic fetal tissues [1–6]. Decreased expression of HLA-G during pregnancy has been noted as a contributing factor to preeclampsia and multiple miscarriages [7–11]. In addition, HLA-G expression has been identified in pancreas, adult thymic cells, and stem cells, as well as in pathological conditions including cancer, transplantation, HIV infection, and inflammatory diseases [12–27]. HLA-G binds to several receptors, including ILT2, ILT4, and KIR2DL4 receptors, to inhibit immune responses of myelomonocytic cells, dendritic cells, T cells, B cells, and NK cells [5, 28–35]. In addition to membrane-bound forms (HLA-G1, -G2, -G3, and -G4), HLA-G is also presented by several soluble isoforms (sHLA-G1, -G5, -G6, and -G7) generated through two mechanisms: alternative splicing and proteolytic release, which is known to be mediated by metalloproteases [36, 37]. Significantly high levels of sHLA-G were determined in several physiological and pathological conditions, including an association with higher pregnancy and implantation rates [8]. It has been determined that a high level of sHLA-G is correlated with clinical manifestation of several diseases, rheumatoid arthritis, systemic lupus erythematosus, asthma, and HIV infection. Increased levels of sHLA-G were correlated with disease progression in patients with hematological malignancies and solid tumors, including patients with acute leukemia, lymphoma, chronic lymphatic leukemia, melanoma, breast cancer, glioma, and renal and lung carcinomas [25, 38]. Recent studies showed that sHLA-G molecules are involved in prolongation of allograft survival in patients with organ/tissue transplantation [39–41]. In most studies plasma/serum levels of HLA-G was determined by ELISA. However, HLA-G-specific ELISA has limitations and does not discriminate the presence of monomer or dimer isoforms of HLA-G. New data has revealed that disulfide-linked dimeric complexes of HLA-G have high preferential binding to immune inhibitory receptors, induce efficient immune inhibitory receptor signaling, and have strong functional activities [32, 42–45]. Limited data are available on the role of HLA-G dimers in clinical pathological conditions. The outstanding questions are whether these HLA-G dimers are formed in patients with organ transplantation and what their function might be in the prolongation of allograft survival. Here we report the analysis of sHLA-G dimers in kidney transplant patients. First, we determined that the levels of sHLA-G dimers were significantly higher in patients with no rejection episode compared with patients that have a chronic rejection of a kidney transplant. The high levels of sHLA-G dimers were associated also with increased expression of the membrane-bound form of HLA-G on monocytes from patients that have no rejection episode of kidney transplant.

HLA-G also has the ability to reduce inflammatory responses through the inhibition of immune cells to produce proinflammatory molecules. One of the potential candidates of such molecules includes matrix metalloproteinase (MMP). Increased expression of MMPs was observed in several human diseases, including cancer and autoimmune diseases, suggesting an involvement of these enzymes in immunity, inflammatory responses, and repair mechanisms. MMP-9 and MMP-2 are especially able to modulate inflammatory responses via cytokine/chemokine actions. Here we demonstrated that the high levels of sHLA-G dimers in kidney transplant patients that have no episodes of rejection were associated with decreased plasma levels of MMP-9. We also determined that the increased levels of sHLA-G dimers linked to the lower levels of proinflammatory cytokines, suggesting the potential role of sHLA-G dimers in controlling the accompanying inflammatory state. From these findings, sHLA-G dimers might be useful as a potential marker to control rejection and the inflammatory status of human kidney allotransplants.

## 2. Materials and Methods

### 2.1. Patients

We enrolled kidney transplant recipients in the study, of which 50 had no evidence of rejection (NR) and 17 had chronic rejection (CR). Kidney function and rejection was evaluated by creatinine level and verified by biopsy.  Table 1 describes the clinical and demographic characteristics of the patient population. Kidney recipients in both categories had similar distribution with respect to gender, age, and race. The distribution of donor source (living or deceased), cold ischemia time, primary cause of renal failure, and immunosuppressive treatment between each group of recipients was not statistically different. Average creatinine levels of CR patients were significantly higher than NR patients (*P* < 0.05). The protocol was approved by the Human Assurance Committee of Georgia Regents University, and written informed consent was obtained from all subjects in the study.

### 2.2. Separation of Human Plasma, PBMCs, and Red Blood Cells from Whole Blood

Blood samples were obtained from patients and collected in EDTA tubes. Aliquots of plasma were stored at −80°C. PBMCs and red blood cells were isolated from buffy coats using Histopaque 1077 (Sigma-Aldrich, St. Louis, MO, USA) gradient centrifugation. Aliquots of PBMCs were stored in liquid nitrogen. Aliquots of red blood cells were stored at −80°C.

### 2.3. Zymography

Total protein of plasma samples was measured using the Bradford method (Bio-Rad, Richmond, CA, USA). Diluted (1 : 50) plasma samples were loaded onto 10% gelatin gels, and electrophoresis was performed at 100 constant voltages. Gels were washed twice with 2.5% Triton-X for 20 min and then incubated at 37°C overnight in zymography Development Buffer (Bio-Rad). The following day, to visualize the bands, gels were stained with Coomassie Brilliant Blue Dye (Bio-Rad) for 3 hrs and destained with Destaining Solution for 45 min. The gels were rehydrated in water overnight, then scanned and analyzed using ImageJ program developed at the National Institutes of Health (USA).

### 2.4. Depletion of Albumin and IgG from Plasma before Immunoprecipitation

Depletion of unwanted proteins that could interfere with the immunoprecipitation of HLA-G from plasma was achieved using ProteoPrep Immunoaffinity Albumin and IgG Depletion kit (Sigma-Aldrich) following the manufacturer's protocol.

### 2.5. Immunoprecipitation of Depleted Plasma and Western Blot Analysis for Detection of HLA-G Monomer and Dimer

100 *μ*L of depleted plasma were mixed with 100 *μ*L of cold RIPA buffer and incubated on ice for 15 min. 20 *μ*L of protein G bead slurry was added to the plasma lysate, then incubated at 4°C for 60 min and centrifuged at 10,000 g for 10 min. 2 *μ*L of MEM-G/9 mAb (Santa Cruz Biotechnology, Dallas, TX, USA) was added to the supernatant and the mixture was incubated at 4°C overnight. After incubation, 50 *μ*L of Protein G bead slurry was added to the plasma lysate, incubated at 4°C for 1 hr, and centrifuged at 10,000 g for 30 sec. 50 *μ*L of Laemmli sample buffer was added to the bead pellet. Samples were run under both reduced and nonreduced conditions. The mixture was denatured at 95°C for 5 min and centrifuged at 10,000 g for 5 min. The supernatant was loaded onto gels for electrophoresis. 30 *μ*L of immunoprecipitated plasma was separated on 10% running gel and 5% stacking gel and transferred to PVDF membrane. The membrane was blocked with 5% BSA and incubated with MEM-G/9 primary mAb, followed by goat anti-mouse IgG-HRP secondary Ab (Santa Cruz Biotechnology). Chemiluminescent HRP-conjugated detection reagent was used for detection. Quantification of blotted proteins was determined by densitometry analysis of scanned films using ImageJ software.

### 2.6. Cytokine and Chemokine Analysis

Cytokine and chemokine plasma levels were measured using the Multi-Analyte ELISArray Kits (Qiagen, Valencia, CA, USA) according to the manufacturer's recommendations. The Array Kits are designed for the simultaneous detection of up to 12 pro- and anti-inflammatory cytokines and chemokines (IL1*α*, IL1*β*, IL2, IL4, IL6, IL8, IL10, IL12, IL17A, IFN*γ*, TNF*α*, and GM-CSF).

### 2.7. Flow Cytometry

PBMCs from both groups of patients (NR and CR) were treated with human TruStain FcX (Fc receptor blocking solution; BioLegend, San Diego, CA, USA) and stained using fluorochrome-conjugated human-specific mAbs against CD3, CD4, CD8, CD14, CD19, and HLA-G. All mAbs were purchased from BD Biosciences (San Jose, CA, USA) or from BioLegend. Cytometry was performed on a cytometer FACSCanto (BD, Franklin Lakes, NJ, USA) and data were analyzed using FlowJo software (Tree Star Inc, Ashland, OR, USA) or Cell Quest software (BD Biosciences). Some results are expressed as percentage of positive cells obtained with specific Ab compared to irrelevant isotype-matched Ab.

### 2.8. Statistical Analysis

Statistical analysis was performed using NCSS (NCSS LLC, Kaysville, Utah, USA) and GraphPad (GraphPad Inc., La Jolla, CA, USA) packages. Normality and continuous numeric data was checked using the Kolmogorov-Smirnoff one-sample test, and comparisons were performed by Student's *t* test or by Mann-Whitney *U* test when appropriate. The *P* value of ≤0.05 was considered to be statistically significant.

## 3. Results

### 3.1. The Levels of sHLA-G1/HLA-G5 Monomer and Dimer Forms Are Increased in Plasma of Nonrejected Kidney Transplant Patients

**Figure 1:**
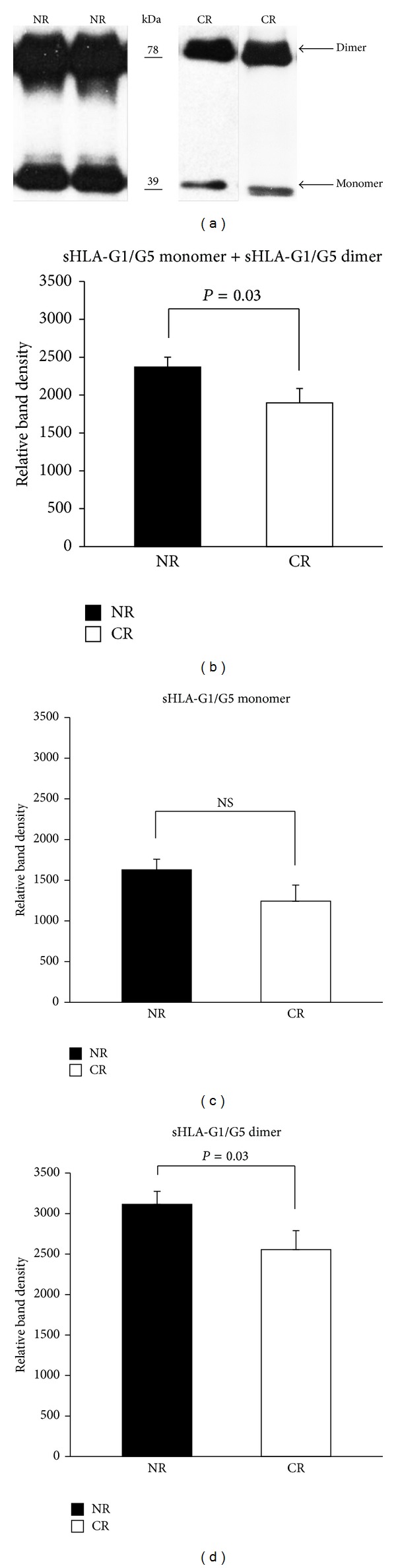
Increased levels of monomer and dimer isoforms of sHLA-G1/G5 in plasma of nonrejecting kidney transplant patients. (a) Plasma from kidney transplant patients was immunoprecipitated with MEM-G/9 mAb. Immunoprecipitates were electrophoresed under nonreducing conditions and Western blot analysis was performed to determine the levels of monomer and dimer of sHLA-G. Representative data from patients with no rejection (NR) and patients with chronic rejection (CR) are demonstrated. Mean relative band density of total sHLA-G (b), sHLA-G monomer (c), and sHLA-G dimer (d) in NR (*n* = 42) and CR (*n* = 17) patients was determined by densitometry. Data are shown as mean ± SEM and analyzed by Student's *t*-test. NS: not significant.

In this study we evaluated the levels of monomer and dimer forms of sHLA-G1 and HLA-G5 isoforms in patients with nonrejected (NR) and chronic rejected (CR) kidney allografts. Immunoprecipitation and Western blot analysis of plasma patients resulted in the discovery of two bands; one corresponding to the expected molecular mass of 39 kDa, which represents the sHLA-G monomer, and one approximately twice that (sHLA-G dimer) (Figure 1(a)). The supernatant from HLA-G5-transfected 721.221 human lymphoblastoid cells was used as a positive control. Both forms of sHLA-G had been determined in the plasma of NR and CR patients, and there was considerable variation in sHLA-G levels within each group. However, the level of total sHLA-G was significantly higher (*P* = 0.03) in NR compared with CR kidney transplant patients (Figure 1(b)). In addition, the level of the monomer form of sHLA-G was slightly higher in the NR group compared with the CR group (Figure 1(c)). We determined that the dimer form of sHLA-G was dominant in the plasma of both groups of patients. Moreover, the level of the dimer form of sHLA-G was significantly elevated (*P* = 0.03) in the NR group of patients (Figure 1(d)). These data indicate that the dimer form of sHLA-G is present and dominates in the plasma of kidney transplant patients.

### 3.2. Increased Expression of the Membrane-Bound Form of HLA-G1 in Monocytes from Nonrejected Kidney Transplant Patients

**Figure 2:**
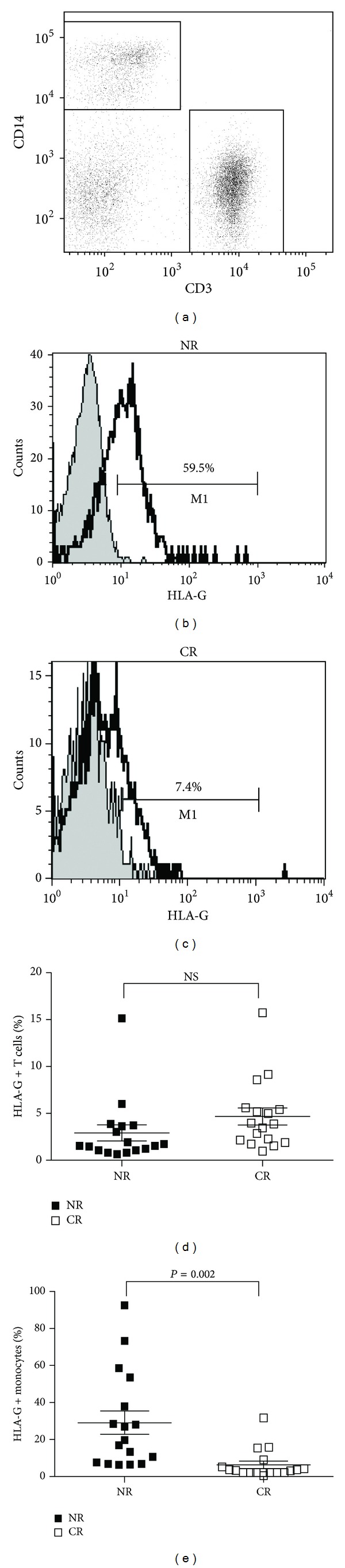
Increased percentages of HLA-G-positive monocytes in nonrejecting kidney transplant patients. (a, b, c) PBMCs from renal allograft patients in both the nonrejected group (NR) and chronic rejected (CR) groups were stained with anti-CD3, anti-CD14, and anti-HLA-G mAbs, and FACS analysis was performed. Representative flow cytometric analysis of PBMCs from indicated groups of renal allograft recipients is shown. (b and c) Histograms shown here were gated on a CD14-positive population. Filled histograms represent the isotype control. Numbers indicate the percentage of HLA-G-positive monocytes. (d) Numbers indicate the percentage of HLA-G-positive T cells in both groups of patients (gated on CD3-positive population). Filled boxes represent data from NR (*n* = 17) and open boxes represent data from CR (*n* = 17) patients. (e) Numbers indicate the percentage of HLA-G-positive monocytes in both groups of patients (gated on CD14-positive population). Data are shown as mean ± SD and analyzed by Student's *t*-test. NS: not significant.

It is known that sHLA-G proteins can be generated by two mechanisms: alternative splicing and proteolytic release, which is mediated by metalloproteases. To determine the potential contribution of the membrane-bound form of HLA-G1 shedding into the pool of sHLA-G molecules in plasma patients, the expression of HLA-G1 on the cell surface of peripheral monocytes and T and B cells has been analyzed in both groups of patients. There was no significant difference in the number of HLA-G1-positive T cells and B cells between NR and CR patients (Figures 2(a) and 2(d) and data not shown). Overall, in both groups of patients, HLA-G1-positive cells represent a small fraction (2–6% of the total) of T and B cells. As expected, the majority of HLA-G1-positive cells in the peripheral blood of both NR and CR patients were determined in the population of monocytes (Figures 2(a), 2(b), and 2(c)). However, the number of HLA-G1-positive monocytes was significantly elevated (*P* = 0.002) in the NR patients, but not in the CR patients (Figure 2(e)). These data revealed that the increased expression of HLA-G1 on monocytes from NR patients might have a substantial contribution to the elevated plasma levels of sHLA-G monomer and dimer forms in those patients.

### 3.3. Analysis of MMP-2 and MMP-9 in Plasma of NR and CR Kidney Transplant Patients

**Figure 3:**
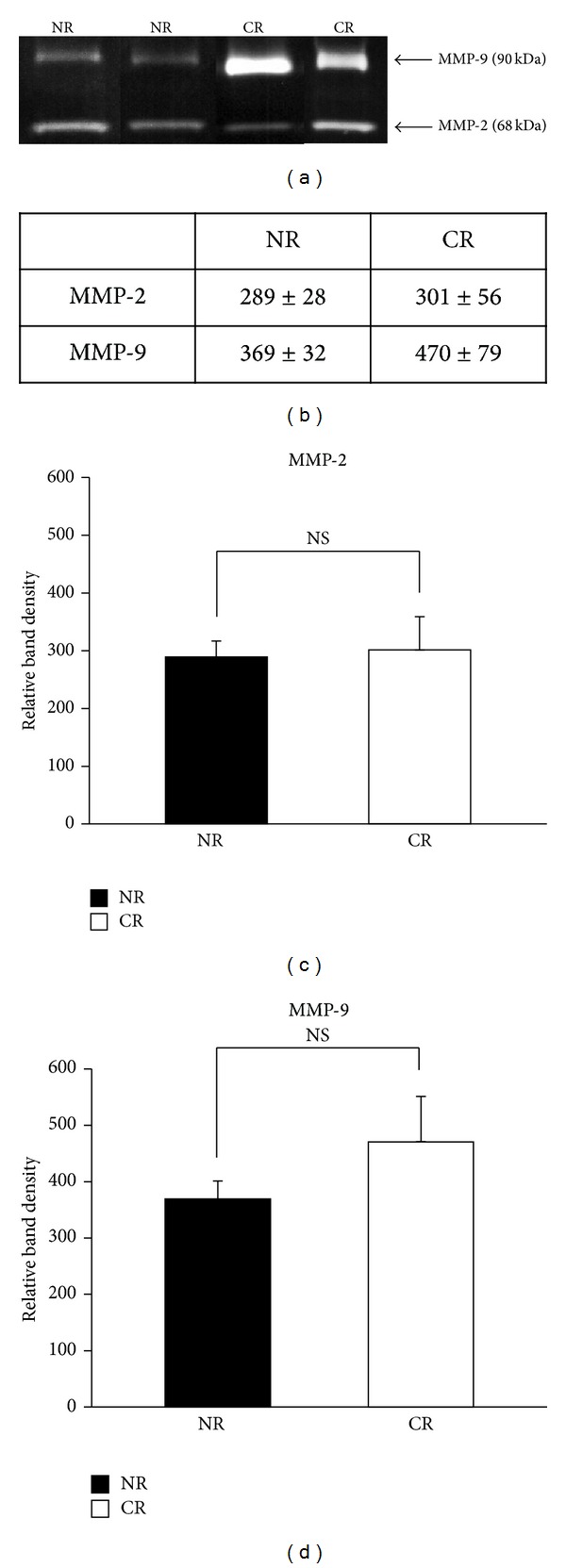
Gelatin zymography of human plasma from kidney transplant patients. Bradford protein assay was used to quantify the amount of plasma needed for zymography that was then loaded onto gelatin gels. The gels were scanned and analyzed using imageJ. (a) Representative gels are of MMP-2 and MMP-9 from patients with no rejection (NR) and patients with chronic rejection (CR). (b) Mean relative band density of MMP-2 and MMP-9 in NR (*n* = 50) and CR (*n* = 17) patients was measured by densitometry. Statistics are shown as mean ± SEM. (c, d) Graphical representation of Figure 3(b). The *P* value was calculated using Student's *t*-test. NS: not significant.

Since one of the mechanisms of the production of sHLA-G involves shedding of the membrane-bound form of HLA-G1 by metalloproteases and since MMPs play a crucial role this class of enzymes, we investigated the levels of MMP-2 and MMP-9 in the plasma of kidney transplant patients. Zymography analysis of transplant patients showed the presence of two bands at 68 kDa and 90 kDa, which correspond to MMP-2 and MMP-9, respectively (Figure 3(a)). As shown in Figure 3(a), plasma from both groups of patients contains substantial amounts of MMP-2 and MMP-9, with no significant difference in the plasma levels of MMP-2 and MMP-9 between NR and CR patients (Figures 3(c) and 3(d)). However, a tendency toward elevation of MMP-9 levels was observed in the CR kidney transplant patients (Figure 3(d)). Since plasma from both the NR and CR groups of patients contains sHLA-G, this data additionally support the possibility that MMPs might play a role in contributing to the pool of total sHLA-G by shedding the membrane-bound HLA-G1 molecules.

Since HLA-G, MMP-2, and MMP-9 are all involved in regulation of the inflammatory response by modulation of cytokines and chemokines and the inflammatory response represents a critical stage in rejection or survival of allogeneic transplants, we next determined the levels of proinflammatory cytokines in kidney transplant patients.

### 3.4. Increased Levels of Proinflammatory Cytokines IL-1*β*, IL-2, and IL-6 in Plasma from Patients with Chronic Rejection of Kidney Transplant

**Figure 4:**
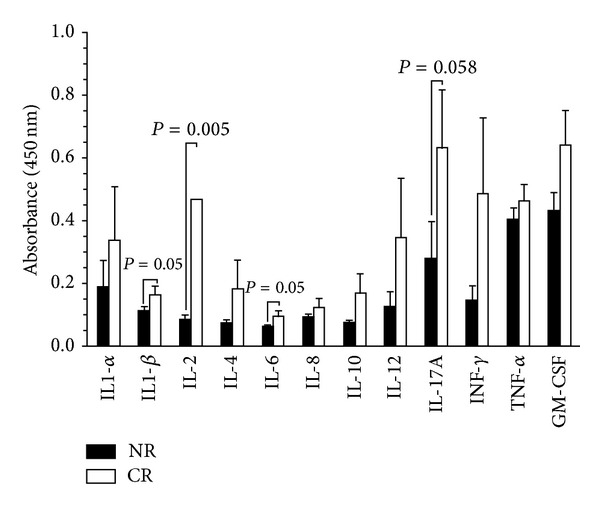
Elevated level of proinflammatory cytokines in plasma of rejected kidney transplant patients. Cytokine and chemokine levels were analyzed using Multi-Analyte ELISArray kits. Cytokine levels from kidney transplant patients with no evidence of rejection (*n* = 9) and with chronic rejection (*n* = 15) are presented as the mean of absorbance values ± SEM. The *P* value was calculated using Mann-Whitney *U* test.

We thus investigated whether the plasma level of proinflammatory cytokines differs between NR and CR kidney transplant patients. For this purpose, we have used a Multi-Analyte ELISArray Kits designed to simultaneously assess the levels of 12 pro- and anti-inflammatory cytokines and chemokines (IL1*α*, IL1*β*, IL2, IL4, IL6, IL8, IL10, IL12, IL17A, IFN*γ*, TNF*α*, and GM-CSF). There was considerable variation in cytokines levels within both the NR and CR patients, especially for IL-1*α*, IL-4, IL-12, and IFN-*γ* (Figure 4). However, the CR kidney transplant patients had significantly elevated levels of proinflammatory cytokines IL-2 (*P* = 0.005), IL-1*β*  (*P* = 0.05), and IL-6  (*P* = 0.05) (Figure 4). In addition, the level of IL-17A was elevated in CR patients. These data support our observation that the dimer form of sHLA-G associates with control of inflammatory responses in kidney transplants patients.

## 4. Discussion

HLA-G is natural molecule involved in the establishment and maintenance of maternal tolerance to fetal semiallogeneic tissues. HLA-G binds to several immune cell inhibitory receptors, for example, ILT2, ILT3, and ILT4, to downmodulate immune responses of myelomonocytic cells, T cells, B cells, and NK cells. Limited polymorphisms, restricted tissue expression, and a relatively restricted peptide presentation make HLA-G a unique molecule, unlike the classical HLA class I molecules. Recently, another unusual characteristic of HLA-G has been discovered: its ability to form a disulfide-linked dimer form both in solution and at the cell surface. HLA-G, unlike most other MHC class I molecules, has two free cysteine residues located in positions 42 and 147 in extracellular domains *α*1 and *α*2, respectively. HLA-G molecules refolded* in vitro* form a disulfide-linked dimer with an intermolecular Cys42-Cys42 disulfide bond [46]. Soluble HLA-G5 molecules expressed by human 293 T cells also form disulfide-linked dimeric and additional oligomeric forms, which can reduce the level of CD8 expression on cytotoxic T lymphocytes (CTLs) [47]. The efficiency of inhibitory signaling is dependent upon several factors, including the stability and avidity of the ligand and its proper structural orientation, which significantly affects the affinity and signaling to targeted inhibitory receptors. Mutagenesis studies of the free cysteines suggested that the HLA-G dimer more efficiently inhibits NK killing than the monomer and increases the efficiency of ILT2 signaling [32, 42, 43, 48]. Experimental data from several groups, including our laboratory, suggest that HLA-G dimer has increased avidity and proper structural orientation to induce efficient inhibitory signaling during ligation with human ILT2 and ILT4, and murine PIR-B-inhibitory receptors [32, 44, 45]. This makes HLA-G dimer as the most powerful ligand form for modulation of inflammatory and alloimmune responses in several pathological conditions, including the prolongation of kidney allograft survival or graft acceptance. Many studies have been designed to determine sHLA-G in the plasma or serum of patients suffering from various diseases. sHLA-G levels were determined in spontaneous miscarriages, in autoimmune diseases, in solid organ transplantation, and in various malignancies. In almost all the studies, the quantification of sHLA-G was analyzed using ELISA. Unfortunately, the available sHLA-G ELISA determines the total amount of sHLA-G protein only, which includes both monomer and dimer forms. Since the dimer represents the most powerful form of sHLA-G, it is very critical to analyze the levels of sHLA-G dimer in healthy and disease conditions. To date, no study has investigated whether HLA-G disulfide-linked homodimers are present in plasma from kidney transplant patients. Here we show that sHLA-G dimers are present in plasma from kidney transplant patients. The levels of sHLA-G dimer were significantly elevated in patients with no rejection episodes compared with patients with chronic rejection, indicating the association of sHLA-G dimers with the prolongation of kidney allograft survival. In support of that, similar levels of expression and percent of immune inhibitory receptor ILT2- and ILT4-positive cells has been determined on monocytes and T and B cells in both groups of patients (data not shown). This clinical finding is in agreement with our previous study using animal models demonstrating that HLA-G dimers prolong the survival of allogeneic skin transplants in ILT transgenic mice ([32, 49, 50] and unpublished data). In the future, it will be important to determine the percentages of sHLA-G1 and sHLA-G5 dimers in total sHLA-G. However, to date, there is no data available demonstrating that sHLA-G1 and sHLA-G5 dimers have different binding and/or different efficiency to induce inhibitory receptor signaling. Our results show that the number of HLA-G1-positive monocytes is significantly increased in NR patients, indicating that the percent of sHLA-G1 dimer might have been elevated within the total level of sHLA-G dimers in plasma of the patients with no episodes of rejection. Recently, Rizzo et al. [37] demonstrated an effective link between MMP-2 and HLA-G1 shedding in 721.221-G transfected cell line, JEG3 cell line, and IL-10-treated PBMCs from five healthy donors using an* in vitro* experiments. However, the process of shedding and especially dimerization of sHLA-G1* in vivo* and in pathological conditions, including kidney transplant patients, could be affected by several factors and requires additional investigation.

The anti-inflammatory effects of sHLA-G dimers represent a new finding for this form of HLA-G. Recently published data by Kuroki et al. [45] demonstrating that in an animal model of collagen-induced arthritis, HLA-G dimer interacting through a murine ILT homologue, the PIR-B receptor exhibited significantly more anti-inflammatory effects compared to monomer. To date, no data is available on studies of the anti-inflammatory effect of sHLA-G dimers in clinical applications. We demonstrate here that an increased level of sHLA-G dimers in kidney transplant patients with no rejection episodes is linked to significantly lower levels of proinflammatory cytokines IL-2, IL-1*β*, and IL-6. It will be important to dissect the mechanisms of sHLA-G dimers controlling inflammatory responses. The anti-inflammatory effect of sHLA-G dimers opens a new strategy to generate useful agents to control inflammatory responses with minimal side effects.

In conclusion, our study shows that sHLA-G dimers are associated with better survival of kidney allografts and control of the accompanying inflammatory response in kidney transplant patients. Thus sHLA-G dimers can be a potential biomarker to control human alloimmune and inflammatory responses.

## Figures and Tables

**Table 1:** Demographic, clinical, therapeutic, and transplant-related parameters of the patients.

	NR	CR
Total, *n *	50	17
Gender, *n *		
Male	14	4
Female	36	13
Recipient age, yr, mean (range)	51.28 (24–75)	47.94 (27–75)
Race, % (*n*)		
Caucasian	26% (13)	29.4% (5)
African American	70% (35)	64.7% (11)
Hispanic	4% (2)	5.9% (1)
Primary cause of renal failure, *n *		
Diabetic nephropathy	15	3
Lupus nephritis	—	3
Renal cystic disease	6	1
Glomerulosclerosis	5	1
Hypertension	11	3
Hypertensive kidney disease	3	2
Other	14	4
Donor type, % (*n*)		
Deceased	80% (40)	70.6% (12)
Living	20% (10)	29.4% (5)
Cold ischemia time, hr, mean ± SD	16.00 ± 10.01 (*n* = 45)	14.57 ± 0.39 (*n* = 13)
Creatinine level, mg/dL, mean (range)	1.48 (0.79–2.56)	2.71 (1.04–6.46)
(HLA-A, B, DRB1) matches, mean ± SD	1.83 ± 0.71 (*n* = 36)	2.08 ± 0.66 (*n* = 13)
(HLA-A, B, DRB1) mismatches, mean ± SD	1.42 ± 0.71 (*n* = 36)	3.62 ± 0.70 (*n* = 13)
Immunosuppressive treatment, *n *		
Azathioprine	2	—
Cyclosporine	5	4
Mycophenolate	30	14
Prednisone	34	13
Rapamycin (Sirolimus)	5	3
Tacrolimus	36	9

NR: no rejection; CR: chronic rejection.
